# The development of speechreading skills in Chinese students with hearing impairment

**DOI:** 10.3389/fpsyg.2022.1020211

**Published:** 2022-11-04

**Authors:** Fen Zhang, Jianghua Lei, Huina Gong, Hui Wu, Liang Chen

**Affiliations:** ^1^Central China Normal University, Wuhan, China; ^2^Shandong University, Jinan, China; ^3^University of Georgia, Athens, GA, United States

**Keywords:** speedreading, hearing impairment, visual speech perception, Chinese, development, cross-linguistic differences

## Abstract

The developmental trajectory of speechreading skills is poorly understood, and existing research has revealed rather inconsistent results. In this study, 209 Chinese students with hearing impairment between 7 and 20 years old were asked to complete the Chinese Speechreading Test targeting three linguistics levels (i.e., words, phrases, and sentences). Both response time and accuracy data were collected and analyzed. Results revealed (i) no developmental change in speechreading accuracy between ages 7 and 14 after which the accuracy rate either stagnates or drops; (ii) no significant developmental pattern in speed of speechreading across all ages. Results also showed that across all age groups, speechreading accuracy was higher for phrases than words and sentences, and overall levels of speechreading speed fell for phrases, words, and sentences. These findings suggest that the development of speechreading in Chinese is not a continuous, linear process.

## Introduction

Effective communication is a multimodal process involving both the ears and eyes (Feld and Sommers, 2009). During this process, the interlocutors not only use their ears to hear speech, but also their eyes to read speech, and then auditory information from the ears and visual sensory information from the eyes need to be integrated into a coherent message (McGurk and Mac Donald, 1976; Rosenblum, 2008; Davies et al., 2009; Heikkilä et al., 2017). While speechreading (also called visual speech perception) typically involves observing the movement of the interlocutors’ lips, jaws and faces, recent studies suggest that even articulatory characteristics such as tongue-back position and intra-oral air pressure are also visible to speechreading (Munhall and Vatikiotis-Bateson, 2004).

While speechreading enhances speech understanding in noisy conditions (Sumby and Pollack, 1954; Alm and Behne, 2015) for persons with both normal hearing (NH) and hearing impairment (HI), the ability to speechread is often critical for persons with HI (Gagné et al., 2006; Tye-Murray et al., 2014). Persons with HI may depend on speechreading to access the spoken language and interact with the hearing world (Kyle et al., 2013). Although the importance of speechreading is well established, the developmental trajectory of speechreading skills is poorly understood and the limited existing studies have produced inconsistent results.

Some studies have revealed some age-related change in speechreading performance (Evans, 1965; Dodd and McIntosh, 1998; Kyle and Harris, 2010; Kyle et al., 2013; Tye-Murray et al., 2014; Chen and Lei, 2017). Dodd and McIntosh (1998) followed a group of 16 deaf children in Australia with severe and profound HI (> 60 dB loss in the better ear across four frequencies of the speech range, 500, 1,000, 2,000, and 4,000 Hz) for 3 years with initial assessment of their speechreading skills when they were 30–57 months. All the children participated in an early intervention program using the total communication approach, i.e., simultaneously signed and spoken English. Speechreading assessments, along with a series of language and cognitive assessments, were conducted at five sessions during the 3-year period using their self-developed Lipreading Assessment for Children with Hearing Impairment (LACHI). The deaf children were found to experience initial increase in speechreading accuracy but then their speechreading ability began to plateau between the ages of 69 months (session 4) and 74 months (session 5). Probably, because the focus of Dodd and McIntosh (1998) was on how early speechreading skills might predict later language development, they provided a combined speechreading score for the initial two sessions without showing the scores for the next three sessions. In addition, although LACHI targeted speechreading of words (e.g., say these words after me: cat, shoe, flower, beautiful, etc.), phrases (e.g., do what I say: push car, horse jump, brush hair, etc.), sentences (e.g., repeat these sentences: I saw a blue car, John did not ask her name, etc.), and conversational speech, it was not clear how speechreading developed at each level or at which level a child’s speechreading skills were plateauing. Hnath-Chisolm et al. (1998) administered the Three-Interval Forced-Choice Test of Speech Pattern Contrast Perception (THRIFT for short) to 44 English speaking children with NH between the ages of 5;7 (Year; Month) and 10;9. THRIFT is basically a version of the odd-man-out task using nonsense syllables. Each of the THRIFT stimuli contains a sequence of three nonsense syllables that are either consonant-vowel (e.g., *voo*) or vowel-consonant pairs (e.g., *eeg*). Two of the syllables in the sequence are the same (e.g., *taw taw*) and one differs from the other two by a single phonologically significant contrast (e.g., *daw*, which differs in initial consonant voicing from *taw*). The odd-man-out may come first, second, or third of the nonsense syllables. The task for the children is to simply select the syllable that is the odd-man-out and tell the experimenter whether it is number 1, 2, or 3. The participants were assigned to three age-bands: 5–7, 7–9, and 9–11 years old, and they completed the test under three conditions: (1) hearing and speechreading combined, (2) hearing alone, and (3) speechreading alone. Testing was completed in a single session and lasted from 45 min to 1 h. For the speechreading alone condition, significant difference was found between the performance of the 5–7 years old (M = 18.44, SD = 9.91) and the 9–11 years old group (M = 28.95, SD = 5.93), but neither of these two groups performed differently from the middle age group (M = 27.14, SD = 7.64). Hnath-Chisolm and colleagues concluded that there was no more development of speechreading abilities after age seven. As can be seen from the speechreading performance of the three groups, the speechreading alone condition of the THRIFT was very challenging for the participants in that the mean score for the speechreading condition was only 24.84 (SD = 9.49) while the mean scores for the hearing alone condition was 84.46 (SD = 14.43) and for the hearing + speechreading conditions was 88.61 (SD = 10.87). In addition, differences in performance between the three age groups were modulated by the function of speech feature contrast tested. Specifically, significant differences in performance as a function of age were only observed in two of the nine speech contrasts, namely, vowel place (e.g., *goo* vs. *gee*;) and final consonant place (e.g., *eeg* vs. *eed*). Evans (1965), Hockley and Polka (1994), Sekiyama and Burnham (2008), and Kyle and Harris (2010), on the other hand, found development in deaf children’s speechreading skills until a tendency to plateau at around age 11 years. Sekiyama and Burnham (2008), for example, used the McGurk paradigm (McGurk and Mac Donald, 1976) to examine the impact of language on the development of auditory–visual speech perception. Their participants included English and Japanese speakers from four age groups: 6-, 8-, and 11-year-old children, and adults. They were asked to identify syllables at various signal-to-noise levels. In the McGurk paradigm, participants are asked to identify syllables audio-only, video-only, and audiovisual presentations. In the audiovisual presentations, the visual syllable may be congruent or non-congruent with the auditory syllable. Both Japanese and English participants showed an increase in accuracy under visual-only condition up to 11 years. No difference was found between the 11-year old and the adults. Yet other studies found development into adolescence. Hockley and Polka (1994), for example, examined the development of audiovisual speech perception in the native speakers of English in Canada. Hockley and Polka also used the McGurk paradigm, and asked 15 adults and 46 children to identify CV syllables /ba/, /va/, /θa/, /da/, and /ga/. The children were divided into four age groups: 5, 7, 9, and 11 years (range = 4;7–12;4). An age-related developmental pattern was found in speech perception in the visual only condition: their visual speech perception skills improved as children grew older. However, only half of the children in the 11-year old group showed an adult-like response pattern. Kyle et al. (2013) examined speechreading development in English speaking deaf and hearing children. The ages of these children ranged from 5 to 14, and were grouped together in 2-year age bands (5–6;11, 7–8;11, 9–10;11, 11–12;11, and 13–14;11). Speechreading at word, sentence, and discourse (short stores) levels was assessed. Results revealed age-related development. Specifically, Kyle and her colleagues found that older children in their study speechread more accurately than the younger children. They also found that speechreading accuracy rate was highest for words, followed by sentences, and lowest for short stories. Tye-Murray et al. (2014) examined the role of age, hearing status, and cognitive abilities in lipreading in school age children (40 with NH and 24 with HI). They used four lipreading tasks, and assessed the children’s perceptual, cognitive, and linguistic abilities. They found age related changes in children from both NH and HI groups. They also found that the group with HI outperformed the group with NH on all four measures of lipreading. Tye-Murray and colleagues concluded lipreading ability in children is not fixed, but rather improves between 7 and 14 years of age. More recently, Moreno-Pérez et al. (2015) and Rodríguez-Ortiz et al. (2017) compared speechreading of phonemes (minimal pairs), words and sentences in three groups of Spanish-speaking adolescents: a group with HI (mean age = 16.4 years, range = 11.7–27.0), a chronological age-matched (CA) group with NH (mean age = 16.3; range = 11.8–27.2) and a younger reading age-matched (RA) group with NH (mean age = 11.7; range = 8.4–16.3). Results showed that the two older groups did not differ from each other, but both groups speechread more accurately than the younger group with NH. While these results suggest some evidence of development in speechreading performance in Spanish children between age 11 and 16, the wide range of participants’ age makes the interpretation less convincing. Regardless, results like these suggest that speechreading skills may continue to develop into adolescence and beyond.

Other studies, however, have failed to find an effect of age on speechreading performance (Alegria et al., 1999; Tremblay et al., 2007; Davies et al., 2009). Tremblay et al. (2007) examined speechreading development in 38 French speakers who were divided into three age groups: 5–9, 10–14, and 15–19. The three groups had similar performances on the visual-only trials in the McGurk task paradigm. They concluded that there was no age-related developmental increase in speechreading performance in French. Ross et al. (2011) also found no improvement in hearing children’ ability to speechread isolated words between the ages of 5 and 14 years.

Thus, as Tye-Murray et al. (2014) have pointed out, the evidence regarding age-related developmental patterns in speechreading is scant and equivocal. The existing studies differed in the level of language analyzed (phonemes, syllables, isolated words, sentences, and stories), the use of real model or videos, the size of the videos, and the use of sound together with the lipreading, the specific task (repetition of the target word, and selection of images, in this last case, the type of distractors; selection of written words), the type of words (familiar vs. unfamiliar words, frequent vs. infrequent words, verbs, nouns, etc.), and/or the type of responses (open-ended vs. forced choice). These studies also differed in whether they were longitudinal or cross-sectional, and in the case of the latter how they classified children into different age groups (e.g., 2-year-interval vs. 4-year-interval age bands). Any of these features could explain the differences in the results, and we will briefly look at the impact of two of these features, test delivery method and the response format. In the study of Dodd and McIntosh (1998), real models were used in that the LACHI was delivered live whereas other studies reviewed here used silent videos. This difference may be important to consider when we evaluate the developmental patterns from different studies of speechreading. Mantokoudis et al. (2013), for example, assessed speechreading skills of 14 deaf adults and 21 users of cochlear implants using the Hochmair Schulz Moser (HSM) sentence test. They found that the speechreading scores obtained with the video presentation mode were statistically lower than a face-to-face communication mode. They suggest that the transmission of speechreading cues over a video screen may lead to lower speech perception scores in comparison to a face-to-face communication mode. Real life presentation of speechreading material may be especially beneficial to individuals with HI (Rönnberg et al., 1983). Mantokoudis and colleagues also found that speechreading performance of their participants was strongly dependent on the individual live model, in particular, the live model’s speaking rate, reinforcing the previously documented differences in intelligibility and speechreadability among different talkers (e.g., Kricos and Lesner, 1985; Bench et al., 1995). Now take a look at the impact of the response method. The study of Dodd and McIntosh (1998) used an open-ended speechreading assessment, and the children had to provide a verbal response to each test item. This may not be as deaf-friendly as the closed-set nonverbal response format (Kyle et al., 2013).

Regardless of the differences in the previous studies, they have pointed to four important issues in speechreading development. First, is there a continuous, linear development (as, e.g., argued for English in Hockley and Polka, 1994) or a lack of such developmental change (e.g., Tremblay et al., 2007 for French). Second, when do children become like adults in their speechreading performance (e.g., sometime after the child’s 6th year as Massaro et al., 1986 suggest, or around 11 years old, or during adolescence). Third, does the linguistic structure of speechreading stimuli itself influence the developmental trajectory of speechreading performance on every level of complexity (Bradarić-Jončić, 1998; Andersson et al., 2001). And last but not the least, is the development of speechreading skills over time language specific? In this study, we explore these possibilities by comparing speechreading words, phrases, and sentences by Mandarin Chinese-speaking children with HI between the ages of 7 and 19 years.

## Materials and methods

### Participants

Two hundred and nine students with HI aged between 7 and 20 years old (mean age = 14.13, SD =4.02) participated in the study. There were 120 males and 89 females. Unaided pure-tone hearing thresholds were measured in the better ear at frequencies of 50, 1,000, and 2,000 Hz, and pure-tone average (PTA) as calculated by averaging the hearing threshold at these three frequency levels. If a student did not respond when a tone was presented at the maximum test level of 100 dB HL, a value 105 dB HL for that frequency was assigned. The mean PTA of the participants was 97.82 ± 12.98 dB HTL. All the students were native speakers of Mandarin Chinese, and at the time of data collection were attending schools in central China. All reported to have normal or corrected-to-normal vision. None had any other impairment, except for HI. None had previously participated in speechreading studies. We obtained informed consent from all participants or, if participants are under 18, from a parent.

Following Kyle et al. (2013), the participants in the present study were also divided into seven 2-year-interval age bands: 7–8 (*N* = 30, with 20 males; mean PTA = 93.11 dB HTL, SD = 11.70); 9–10 (*N* = 19, with 13 males; mean PTA = 96.75 dB HTL, SD = 10.78); 11–12 (*N* = 23, with 12 males; mean PTA = 99.49 dB HTL, SD = 8.75); 13–14 (*N* = 36, with 17 males; mean PTA = 100.33 dB HTL, SD = 10.04); 15–16 (*N* = 31, with 16 males; mean PTA = 100.00 dB HTL, SD = 14.83); 17–18 (*N* = 33, with 19 males; mean PTA = 97.52 dB HTL, SD = 14.24); and 19–20 (*N* = 37, with 23 males; mean PTA = 97.15 dB HTL, SD = 14.19). One-way ANOVA revealed no significant difference in their severity of hearing loss, *F*(6,208) =1.14, *p* > 0.05.

The percentage of hearing aid users in each group is largely similar, except for the 7–8-year-old group with 26 hearing aid users and only four non-hearing aid users. This information is included in Table 1 together with other demographic information of the participants. All participants completed the Chinese version of Raven’s Standard Progressive Matrices (Zhang and Wang, 1989). Raven’s Standard Progressive Matrices (RSPM) was designed to measure a person’s ability to form perceptual relations and to reason by analogy, and it is broadly used as a nonverbal test that measures general intelligence. None of the participants scored below the 5th percentile for their age group. One-way ANOVA was used to compare Raven’s scores of different HI groups. The results showed a significant group effect, *F*(6, 202) = 127.29, *p* < 0.001. *Post hoc* tests (Bonf) indicated that participants in the 11–12-year-old group scored higher than the 9–10-year-old group (*p* < 0.001), those in the 13–14-year-old group scored higher than the 11–12-year-old group (*p* < 0.001), and the 15–16-year-old group scored higher than the 13–14-year-old group (*p* < 0.001). No statistically significant differences were observed between the 7–8-year-old group and the 9–10-year-old group, or among the three oldest groups (*p*s > 0.05).

**Table 1:** Study population demographics.

	Groups						
	7–8 years	9–10 years	11–12 years	13–14 years	15–16 years	17–18 years	19–20 years
Age (SD)	7.70 ± 0.47	9.63 ± 0.50	11.61 ± 0.50	13.53 ± 0.51	15.55 ± 0.51	17.67 ± 0.48	19.46 ± 0.51
Gender (F/M)	10/20	6/13	11/12	19/17	15/16	14/19	14/23
Hearing loss (SD)	93.11 ± 11.70	96.76 ± 10.78	99.49 ± 8.75	100.33 ± 10.04	100.00 ± 14.84	97.52 ± 14.24	97.15 ± 14.19
Hearing aid User (Yes/No)	26/4	10/9	9/14	14/22	13/18	13/20	14/23
Raven (SD)	17.53 ± 9.08	19.55 ± 6.25	26.91 ± 3.08	37.64 ± 3.85	43.55 ± 7.15	46.67 ± 4.39	45.68 ± 4.78

### Material

**Figure 1:**
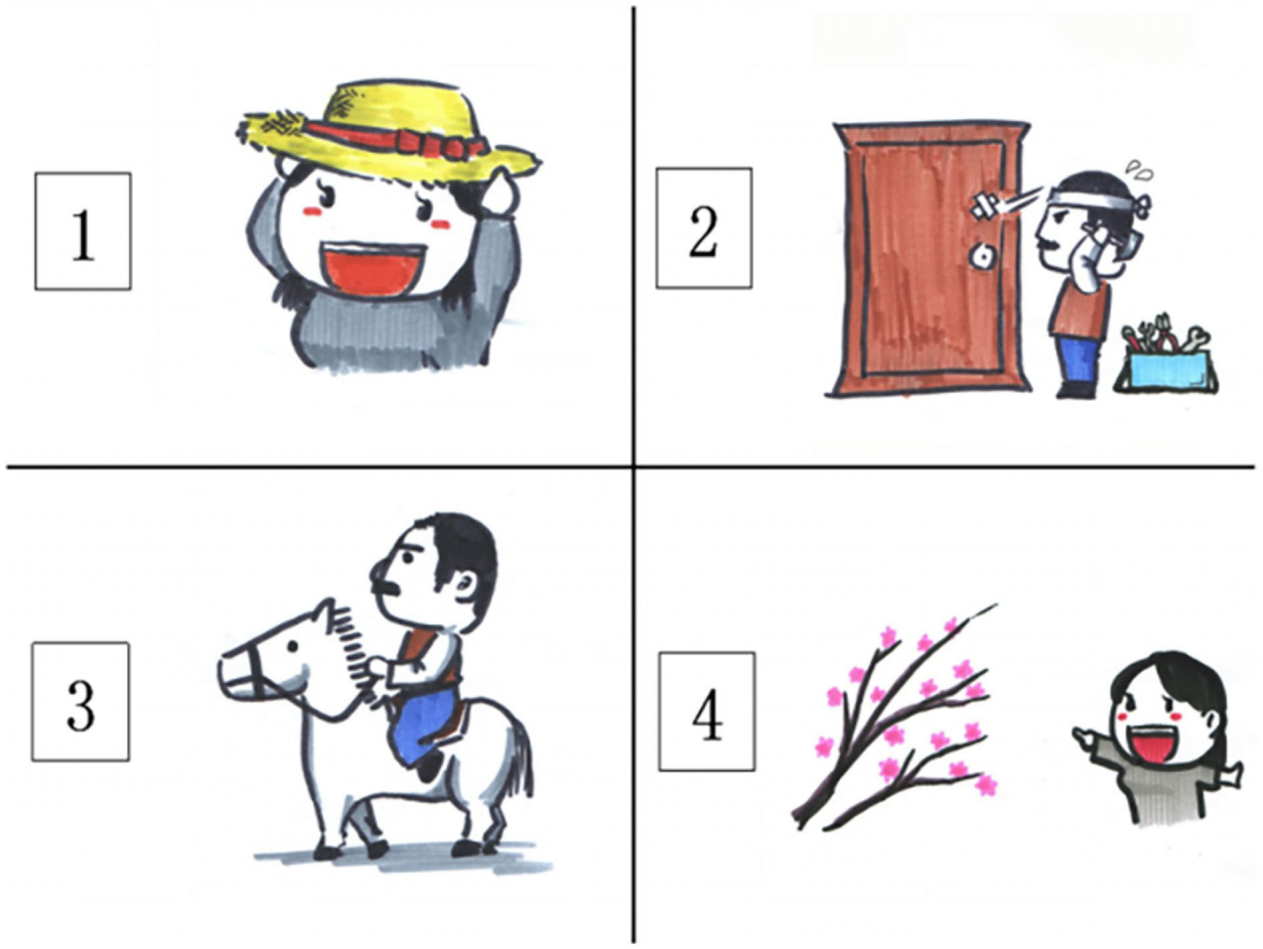
Illustration for response screen of the sentence subtest.

The computer-based Chinese Speechreading Test (CST, Lei et al., 2019) was adopted to assess participants’ speechreading skills. The CST followed the recommendations of Kyle et al. (2013), and is a computer-based speechreading test with the video-to-picture matching design. It consists of three subtests targeting three different linguistic levels. Each subtest has 12 test items, and each test item is associated with a silent video chip of a male Chinese speaker saying that particular item (either a word, a phrase, or a sentence). The word subtest consists of 12 single character target words (henceforth *word* for short). For each target word, there are three distractors are related to the target in terms of visemic properties, that is, they share the initial viseme but differ in the final viseme. For example, the distractors for the target word 笔(bi3,“pen”) are 杯(bei1,“cup”), 饼(bing3,“cookie”), and 表(biao3,“watch”). The phrase subtest consists of 12 target phrases that are two-character words. Each of the phrases (target phrases and the distractors) contains one of the target words from the words subtest, and the words in the words subtest are the second word in the phrases subtest. For example, when the target phrase is 铅笔(qian1bi3,“pencil”), the distractors are 水杯(shui3-bei1,“water cup”), 月饼(yue4bing3,“moon cake”), and 手表(shou3biao3,“wrist watch”). The sentence subtest consists of 12 simple transitive sentences. Each sentence is five-word long, with a two-word phrase as the subject and another two-word phrase as the object. In addition, the object phrase always comes from the phrases subtest. To keep the subject of the sentences from contributing spuriously to any difficulty in speechreading, we used high-frequency phrases referring to common relationships or descriptors of people, such as 姐姐(jie3jie3,“elder sister”), 妹妹(mei4mei4,“younger sis-ter”), 男孩(nan2hai2,“boy”), and 女孩(nü3hai2,“girl”). For example, one target sentence is 叔叔骑白马(shu1 qi2 bai3 ma3, “a man rides a white horse”), and the distractors are 阿姨戴草帽(a1 yi2 dai4 cao3 mao4, “a woman wears a straw hat”), 叔叔修木门(shu1 xiu1 mu4 men2, “a man repairs a wooden door”), and 阿姨赏腊梅(a1 yi2 shang3 la4 mei2, “a woman looks at winter sweet flower”). This is illustrated in Figure 1.

After the presentation of the video clip of the target word (or phrase, or sentence), an array of four pictures (one target and three distractors) will be presented, and the participant must choose the picture that best matches the target item that he or she has seen in the video clip. To minimize the effect of the position on speechreading performance, the position of the pictures for the target items was counter-balanced. The internal reliability of the CST, calculated through Cronbach’s alpha, was 0.86 (for details of CST, see Lei et al., 2019).

### Procedure

E-prime 2.0 software package (Psychology Software Tools, Schneider et al., 2002) was used to administer the speechreading tasks on a PC. Participants were tested individually on a computer, and the order of the three subtests was counterbalanced across participants. Instructions in Mandarin Chinese were displayed on the computer screen, and the participants pressed designated buttons on the keyboard to select the picture that would match the target. Participants were allowed to ask for clarification using their preferred mode of communication (e.g., Chinese sign language or written Mandarin Chinese).

After participants watched the silent video clip of the male speaker producing a target item, they were presented with a response screen showing four pictures numbered from 1 to 4. They would type one unique number from 1 to 4 to select picture of the target item. As soon as a response screen displaying the picture sets showed up, E-prime started to track the reaction time automatically. Each item was presented only once, and no feedback was provided to the participants during the tests. Participants were instructed to complete the tasks as accurately and as quickly as possible, but they were given no time constraint. Participants were told that they could type their guesses if they were uncertain about a test item. The experimenter monitored the participants throughout the tests to ensure they were completing the tasks correctly and independently. The tasks took each participant about 20 min to complete.

## Results

Table 2 summarizes the descriptive statistics of the dependent variables of interest, namely, the mean accuracy rate (% correct) and mean response time (RT in seconds), according to age group and linguistic level. Data analysis was performed using a 7(age group) × 3(linguistic level) ANOVA, where age group (7–8, 9–10, 11–12, 13–14, 15–16, 17–18, and 19–20) was the between-subjects factor, and linguistic level (word, phrase, and sentence) was the within-subjects factor. The analysis was done separately for the two dependent variables.

**Table 2:** Accuracy rate (% correct) and mean response time (RT in seconds) according to age group and linguistic level.

Age groups	N	Word		Phase		Sentence	
		Accuracy	RT	Accuracy	RT	Accuracy	RT
7–8	30	0.47 ± 0.21	6.87 ± 2.99	0.58 ± 0.26	6.48 ± 2.12	0.43 ± 0.22	7.17 ± 2.94
9–10	19	0.56 ± 0.19	5.23 ± 2.32	0.65 ± 0.19	4.38 ± 1.61	0.55 ± 0.19	5.84 ± 2.70
11–12	23	0.55 ± 0.17	5.34 ± 2.48	0.66 ± 0.17	4.78 ± 1.98	0.53 ± 0.22	6.02 ± 2.99
13–14	36	0.49 ± 0.18	5.76 ± 2.66	0.64 ± 0.21	5.70 ± 2.80	0.51 ± 0.23	6.96 ± 2.59
15–16	31	0.40 ± 0.17	5.40 ± 2.40	0.55 ± 0.23	5.31 ± 2.07	0.40 ± 0.22	6.09 ± 1.98
17–18	33	0.39 ± 0.16	5.77 ± 2.38	0.48 ± 0.20	5.74 ± 2.45	0.37 ± 0.21	6.24 ± 2.32
19–20	37	0.41 ± 0.18	6.31 ± 2.33	0.50 ± 0.21	5.74 ± 2.13	0.44 ± 0.22	6.60 ± 2.62

### Accuracy rate

A 7(age group) × 3(linguistic level) ANOVA was used to investigate differences in the mean accuracy rates, which are plotted in Figure 1. Results revealed a significant main effect of age, *F*(6, 202) = 4.31, *p* < 0.001, η_p_^2^ = 0.11. *Post hoc* test (Bonf) indicated that participants from groups 9–10, 11–12, and 13–14 speechread significantly more accurately than those from group 15–16, *p* < 0.001, no other group difference in speechreading accuracy was found (*p* > 0.05). The main effect of linguistic level was also significant, *F* (2,404) = 55.55, *p* < 0.001, η_p_^2^ = 0.22. *Post hoc* test (Bonf) indicated that the speechreading accuracy for phrases was significantly higher than that for word and sentence (*p* < 0.05 for both comparisons). No significant interaction was found between age group and linguistic level, *F* (12,404) = 0.81, *p* > 0.05.

### Response time

**Figure 2:**
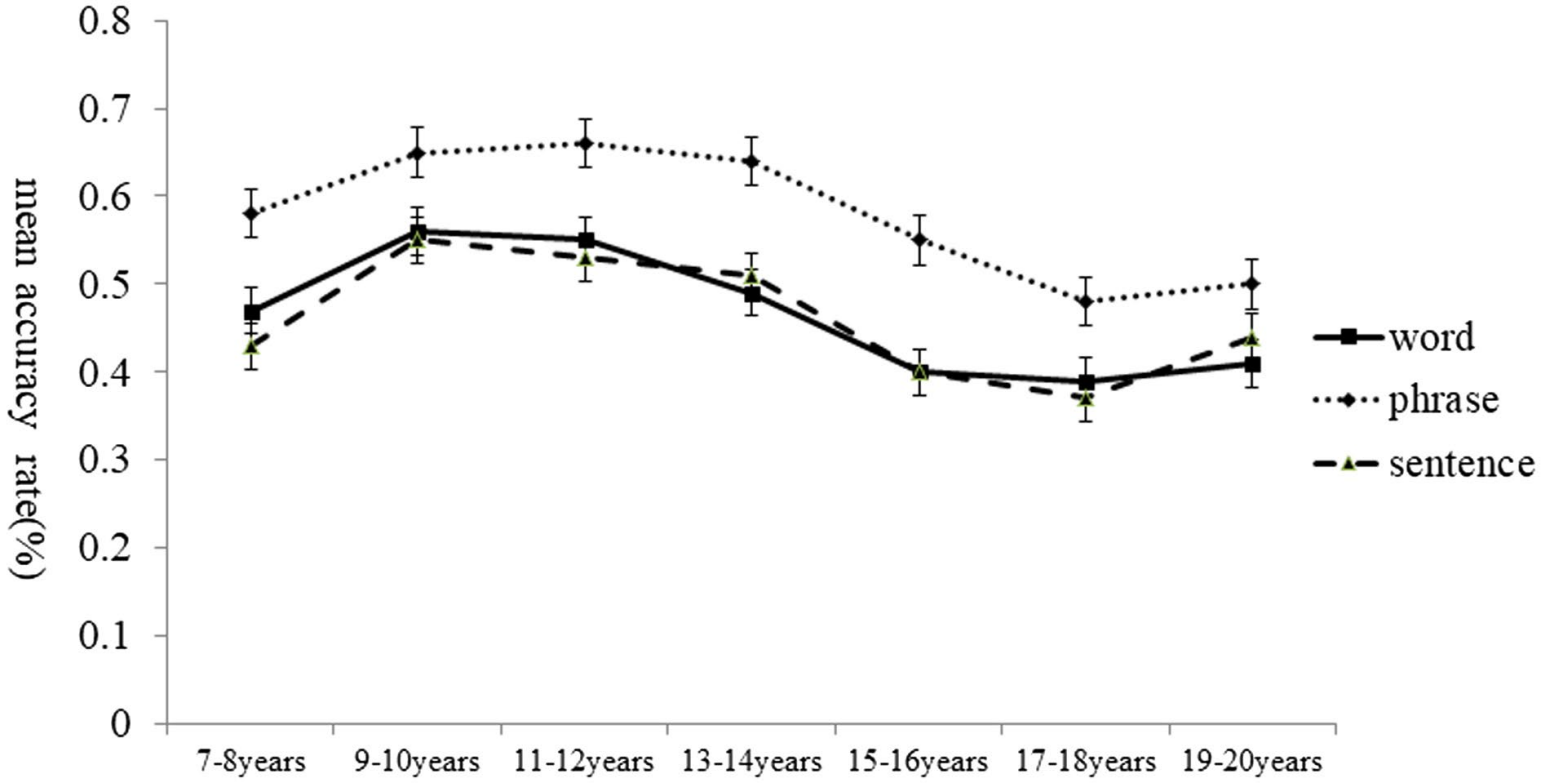
Mean accuracy rates of speechreading in Chinese as a function of age group and linguistic level.

**Figure 3:**
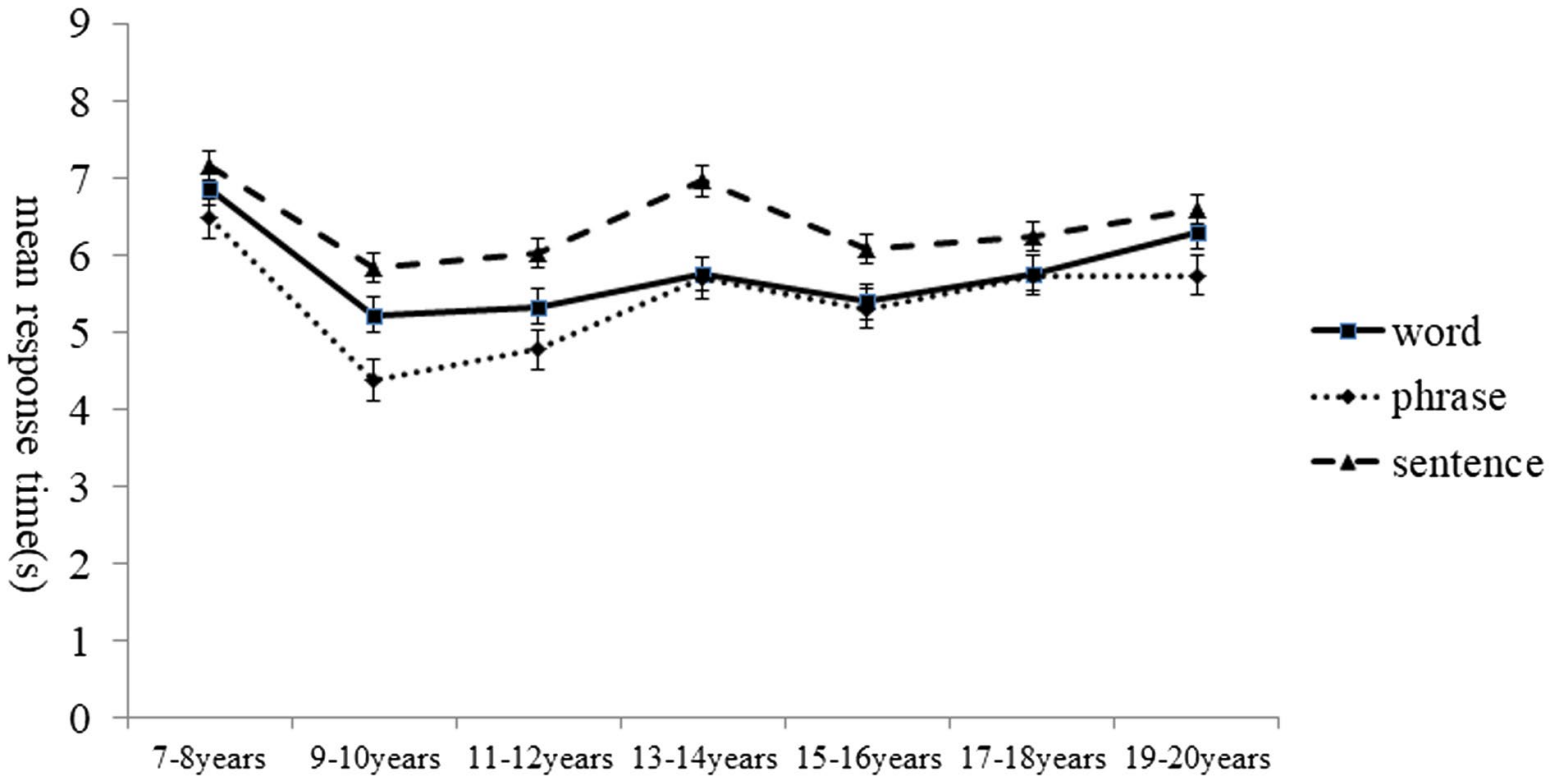
Mean response time of speechreading in Chinese as a function of age group and linguistic level.

A separate 7(age group) × 3(linguistic level) ANOVA was used to investigate developmental differences in the mean response times in second. The response time represents the time lapse prior to responding to the items of different linguistic levels, and the mean response times are plotted in Figure 2. Results revealed no main effect of age, *F*(6,202) = 1.93, *p* > 0.05, but the main effect of linguistic level on response time was significant, *F*(2,404) = 19.42, *p* < 0.001,and η_p_^2^ = 0.09. *Post hoc* tests (Bonf) indicated that the response time for phrases was shorter than that for sentences, and the response time for sentences was higher than that for word. The ANOVA results revealed no significant interaction between the linguistic level and the age group, *F* (12,404) = 0.64, *p* > 0.05 (Figure 3).

## Discussion

The main objective of the study is to enhance our understanding of the developmental trajectory of speechreading in Chinese students with HI. Three findings are highlighted. First, speechreading performance did not improve significantly between 7 and 14 years of age. This result is consistent with what Ross et al. (2011) have reported for speechreading isolated words by typically developing English speaking children. Jerger et al. (2009) also reported similar findings for English-speaking children with HI aged 5–12 who had no significant change in the speechreading ability as they grew up. This finding, however, is contrary to what Kyle et al. (2013) and Tye-Murray et al. (2014) have reported for English-speaking children with HI and NH. These different results raise more questions than answered. Are the different results due to different measures used, or due to the different levels of the sensitivity of the measures? Is it possible at all to develop a speechreading measure that can meaningfully compare the development of speechreading in different languages? Regardless, these different results raise the need to investigate the factors that contribute to these different results. Efforts to resolve these different results will also have implications on speechreading training. Recent studies show that speechreading by 4–5 year old hearing children can be improved by 3 weeks of computerized speechreading training (Buchanan-Worster et al., 2021). Will speechreading training be similarly effective for children aged between 7 and 14 years of age even if they are not going to experience any developmental changes during these years?

Our second finding is that the speechreading ability in Chinese HI students started to decline, as evidenced by the less accurate performance among the three oldest groups aged 15–16, 17–18, and 19–20. The observation regarding the decline of speechreading ability in Chinese students with HI from the age of 15 is different from what Tremblay et al. (2007) found for the three groups of French-speaking participants (5–9, 10–14, and 15–19 years old) who had similar performances on the speechreading task. However, our second finding is consistent with the results of Chen and Lei (2017) who found 13-year-old Chinese students with NH speechread vowel sounds more accurately and quickly than the 16-years-old. Chen and Lei suggest that this decline in the 16-year-old may be related to their decreasing dependence on the spoken language and accordingly their increased reliance on the written language. We concur with this explanation on the basis of two observations. Both observations relate to the close relationship between reliance on the oral channel and speechreading performance. On the one hand, Auer and Bernstein (2008) found that their deaf participants who relied more on the spoken channel for word learning also had superior speechreading performance. On the other hand, Strelnikov et al. (2009) found that after the reception of a cochlear implant tendency their deaf participants tended to see an improved performance in speechreading of words and phonemes in French. In other words, as the deaf participants increased reliance on the spoken channel following cochlear implantation, they also demonstrated significant improvement in speechreading skills. Both observations point to a close relationship between reliance on the spoken channel and speechreading performance. The older the participants are in the present study, the more demand they would experience for reading written material, particularly at the start of high school. The highly competitive college entrance examination in China may have forced the participants in the present study to spend increasingly more time studying the textbooks of different subjects alone. As a result of the increased attention to written material, the older participants in the present study also experienced a decline in speechreading accuracy. Regardless, this pattern of retraction makes it even more difficult to answer the question of when children become like adults in their speechreading performance (e.g., sometime after the child’s sixth birthday as Massaro et al. (1986) suggest, or around 11 years old, or during adolescence).

A third finding relates to the effect of linguistic complexity on speechreading performance. Participants across all age groups speechread phrases more accurately than words and sentences, and overall levels of speechreading speed fell for phrases, words, and sentences. These results appear to contradict the well-known effect of linguistic complexity on speechreading. In particular, studies of speechreading in other languages have generally found that when the complexity and length of the linguistic unit increased, speechreading performance would decrease (Green et al., 1981; Lyxell and Holmberg, 2000; Kyle et al., 2013). For example, English-speaking children with HI and those with NH both were found to be most accurate at speechreading single words, followed by sentences and then by short stories. Lei et al. (2019) suggest that languages may differ in the measures of complexity and length of linguistic units due to some language-specific properties. The words and the phrases used in the CST differ in two important ways. In terms of form, words are monosyllabic (e.g., 衣, yi1, “clothes”) whereas phrases are disyllabic (e.g., 毛衣, mao2 yi1, “sweater”). So at the surface, words in Chinese may contain less semantic content and provide less opportunities for analysis during speechreading. In terms of meaning, words may be more ambiguous and have more potential referents than phrases, because for example the referent of 铅笔, qian1bi3, “pencil” is a subset of the referents of 笔, bi3, “pen, pencil, or any writing instrument.” These two differences may have caused phrases in the present study to be less complex than the words as far as speechreading is concerned. It should also be noted that while we found differences in speechreading performances at the three linguistic levels, the levels of complexity of the speechreading material was not found to influence the developmental trajectory of speechreading performance (Bradarić-Jončić, 1998; Andersson et al., 2001). The seemingly counterintuitive finding that phrases (two-character words) in the present study are more difficult to speechread than single character words is consistent with recent studies on character reading and word reading in Chinese. Specifically, there is ample evidence that Chinese-speaking children performed significantly better on reading the same characters when embedded within words than when alone (Wang and Mcbride, 2016). This finding can be accounted for by the Neighborhood Activation Model (NAM; Luce and Pisoni, 1998). The NAM was originally proposed as a theoretical model of auditory word recognition based on the statistical properties of the spoken language. It has since been applied to speechread silent English (Auer and Bernstein, 2008). According to the NAM, words in the mental lexicon are organized into similarity neighborhoods, and word recognition requires the selection of the target word from its competing lexical neighbors. Some words come from sparse neighborhoods and have few neighbors, whereas other words come from dense neighborhoods with many neighbors. During the process of word recognition, the activation of neighbors may interfere with the processing of the target words. The NAM predicts that words with high neighborhood density will be the harder to recognize than words with low neighborhood density. In our study, ‘phrases’ involve one more character (which is itself a morpheme) than the single words, and are less likely to be confused with other lexical items (‘low neighborhood density’). By contrast, monosyllabic word items in the present study may activate a larger range of other words and accordingly have higher lexical neighborhood density, and as a result they may be recognized less quickly and accurately. While the NAM was originally proposed for alphabetic languages such as English, several studies have attempted to examine its application to Chinese (Wang et al., 2010; Liu et al., 2011; Wang et al., 2014). While results from these studies do not all support the NAM (e.g., Wang et al., 2010), they have all found that Chinese word recognition scores are higher among disyllables than among monosyllables. For example, Liu et al. (2011) examined the lexical neighborhood effect on spoken-word recognition in ninety-six Chinese speaking children with NH (age ranged between 4.0 and 7.0 years). The test items included six lists of monosyllabic and six lists of disyllabic words (20 words/list), and were further divided into “easy” and “hard” halves according to the word frequency and neighborhood density based on the theory of Neighborhood Activation Model (NAM). The children were divided into three different age groups of one-year intervals. Results showed that children scored higher with disyllabic words than with monosyllabic words, and the word-recognition performance also increased with age in each lexical category. Thus, the results from the study of Liu et al. (2011) showed that neighborhood density influenced the performance in Chinese word recognition. Similar results were also reported in Wang et al. (2014) for both children and adults with NH. We are in the process of creating speechreading material with the test items from Liu et al. (2011) and Wang et al. (2014) in order to confirm the different speechreading performances in words and phrases discovered in the present study, and also to formally test whether the NAM model applies to speechread silent Chinese in children with and without HI.

While these results provide valuable contribution to a better understanding of the development of speechreading in Chinese students with HI, these initial findings need to be interpreted carefully. One important consideration for future studies is that we were unable to consider various factors that may contribute to speechreading performance, such as the participant’s general oral language proficiency, vocabulary skills, working memory, duration of hearing aid use, speech intelligibility, phonological skills, and reading skills.

## Conclusion

The present study explored the development of speechreading of words, phrases, and sentences in Chinese speakers with HI. We did not find any age-related development between ages 7 and 14, but we found significant decline around 14 years of age. From a crosslinguistic perspective, our results seem to argue against a continuous, linear development model (as, e.g., suggested for English in Hockley and Polka, 1994), but instead are more compatible with the lack of developmental change (as was documented for French in Tremblay et al., 2007). Our results raise the possibility that the development of speechreading skills over time may be language specific. Unfortunately, information on speechreading development is only available for a limited number of languages. It is necessary to study speechreading in a typologically diverse set of languages from different language families for building a crosslinguistic model of speechreading development. Another fruitful area of future research is the application of maximally comparable designs and tasks for meaningful cross-linguistic comparisons of speechreading development.

## Data availability statement

The original contributions presented in the study are included in the article/supplementary material, further inquiries can be directed to the corresponding author/s

## Ethics statement

The studies involving human participants were reviewed and approved by the Central China Normal University Ethics Committee (IRB#CCNU-IRB-20190102). Written informed consent to participate in this study was provided by the participants’ legal guardian/next of kin.

## Author contributions

ZF and JL designed and implemented the research and drafted the manuscript. ZF, JL, and LC contributed to the analysis and interpretation of the data. LC revised the manuscript. ZF, HG, and HW carried out the experiment and collected the data. All authors contributed to the article and approved the submitted version.

## Funding

This study receieved funding from the China National Education Sciences Planning Foundation [Grant #CBA220315] and from Central China Normal University’s support of Research on the Construction and Application of Chinese speechreading Corpus [CCNU22LM004].

## Conflict of interest

The authors declare that the research was conducted in the absence of any commercial or financial relationships that could be construed as a potential conflict of interest.

## Publisher’s note

All claims expressed in this article are solely those of the authors and do not necessarily represent those of their affiliated organizations, or those of the publisher, the editors and the reviewers. Any product that may be evaluated in this article, or claim that may be made by its manufacturer, is not guaranteed or endorsed by the publisher.
